# Predictors of Sports Gambling among College Students: The Role of the Theory of Planned Behavior and Problem Gambling Severity

**DOI:** 10.3390/ijerph18041803

**Published:** 2021-02-12

**Authors:** Xin Wang, Doyeon Won, Hyung Sang Jeon

**Affiliations:** 1 College of Physical Education, Liaocheng University, Liaocheng 252059, China; wangxin900118@naver.com; 2 Department of Kinesiology, Texas A&M University—Corpus Christi, Corpus Christi, TX 78412-5820, USA; 3 Department of Taekwondo, Yong In University, Yongin-si 17092, Korea

**Keywords:** sport gambling, problem gambling, theory of planned behavior, sport betting

## Abstract

The current study investigated what influences college students’ behavioral intention and behavior towards sports gambling using the theory of planned behavior (TPB) as a theoretical framework. The study also explored the moderation effect of problem gambling severity in the relationships between TPB determinants, behavioral intention, and sports gambling behavior. Data were collected from 334 college students from four different universities in the U.S. and analyzed through partial least squares structural equation modeling (PLS-SEM) and multi-group analysis. The results indicated that attitude was the most critical determinant of college students’ sports gambling intentions, followed by the subjective norms, while both behavioral intention and perceived behavioral control were significant predictors of sports gambling behavior. The study also found some meaningful moderation effects of problem gambling severity. Subjective norms were influential on college students with greater problem gambling severity, while attitude was the strongest predictor of recreational sports gamblers. Suggestions on prevention and treatment programs regarding sports gambling and problem gambling are discussed.

## 1. Introduction

Given the lasting influence of problem gambling and the increase in the size of the sports gambling industry, it is critical to understand what influences college-aged individuals’ sports gambling behavior, especially given the negative personal, societal, economic, and health ramifications of problem and at-risk gambling [1,2,3]. While most of those who gamble or bet on sports participate in gambling activities for light entertainment and socialization, some become involved in gambling activities too much and become addicted to gambling. According to the National Council on Problem Gambling, problem gambling or gambling addiction is defined as “all gambling behavior patterns that compromise, disrupt or damage personal, family or vocational pursuits” [3]. It is well documented that problem gambling can cause various personal and social issues, including psychological disorders, relationship stress, low self-esteem, and financial distress [4,5].

The U.K. National Health Services (NHS) found from the ‘Health Survey for England 2018′ that the number of problem or pathological gamblers registered at 0.5%, but as many as 4% (of those questioned) identified as “at-risk” gamblers, more than double the figure recorded in 2015 [6]. Especially, college-aged populations are at higher risk for problem gambling. For example, every one in four displayed signs of a gambling problem among men between the ages of 18–24 [7]. A recent meta-analysis, based on 72 studies conducted between 1987 and 2016 surveying 41,989 college students world-wide, revealed that 6.13% of the surveyed students were probable pathological gamblers while 10.23% were problem gamblers [5].

For college students in the U.S., betting on sports is a well-received gambling activity (e.g., March Madness bracket pools; daily fantasy sports, playing pickup basketball for money) due to the importance of sports in American culture. North American professional sports leagues are in favor of legalizing sports gambling in the United States. For example, the National Basketball Association (NBA) commissioner claimed that it is inevitable to face expanded legalized sport betting in the U.S., and now the league plans to seek a 1% cut of all sports wagers [8]. As many as 22 out of 50 states in the U.S. have legalized or are planning to legalize sports betting, including online betting.

Thus, it is plausible to expect that college students will be likely to be exposed to a greater level of chances to participate in various sport betting activities, and, consequently, some of them may get addicted to sports gambling. In countries like the U.K., where sports gambling has been established for a long while, there are more gambling problems at the individual and societal levels, especially with sports-based gambling activities. For instance, according to the Gambling Commission in the U.K., 47% of respondents over the age of 16 participated in at least one form of gambling each month while sports gambling was the most popular form of gambling activities, such as in-play sports betting, horse races, and football pools, for them [9].

Therefore, understanding what influences college students’ sports gambling behavior is important for public health and education agencies. Consequently, the current study investigated what predict college students’ sports gambling behavior using the theory of planned behavior (TPB) [10]. More specifically, the study also compared the differences between recreational and problem gamblers.

### 1.1. Gambling among College Students

According to the 2001 Harvard School of Public Health College Alcohol Study (CAS), 47% of student-athletes, 47% of sports fans, and 38% of other students (those who were neither a student-athlete nor a sports fan) gambled during the past school year [11]. The CAS also revealed that, among male college students, almost 33% of athletes, 32% of sports fans, and 18% of other students had some ‘sport’ gambling experiences. Not surprisingly, ‘male’ college students interested in sports and attended a college with high ‘sport interest’ were more likely to gamble on sports, use a ‘bookie’, and gamble on the Internet [11].

People with gambling problems typically begin their gambling activities during their mid-20s, which persists for about 10 years. Whereas those with pathological gambling are likely to bet on sports (e.g., in-play betting, office sports pool, sports gambling at a casino or a bookie) more often than any other types of gambling activities [12]. In other words, sports gamblers are exposed to a higher level of risk for experiencing gambling-related problems than those participating in other types of gambling activities [1,12]. In addition, gambling and substance abuse overlap among college students, while betting on sports is a popular gambling option for college students [13]. A longitudinal study with young adults in the United States found that gambling frequency is significantly associated with substance use and risks, such as alcohol use disorder and other illegal drug addictions [13].

One of the popular betting activities includes ‘daily fantasy sports’ (DFS) in relation to sports gambling. While some controversies exist regarding whether fantasy sports contests are a form of gambling activities, playing fantasy contests with entry fees can be considered a form of gambling [1,14]. More recently, the U.S. Internal Revenue Service (IRS) concluded that DFS is a form of gambling because the ‘skill’ involved in selecting and trading fantasy players is comparable to the skills involved in choosing winders of other sports gambling activities [15]. A recent study found that a higher level of college students’ involvement in fantasy sports is significantly related to the likelihood and frequency of gambling [1]. Especially, college students who pay an entry fee to play fantasy sports are likely to have mental health issues compared to those who only play free-to-play fantasy sports [1]. While there are personal and business-related utilities of fantasy sports, playing fantasy sports have its own negative consequences. Sports gambling, especially fantasy sports, might be more appealing to younger individuals who are less prepared to deal with gambling activities’ negative outcomes [14]. Similar to Martin et al.’s findings [1], Houghton et al. reported that pay-to-play (P2P) fantasy sports participants are related to higher levels of sports betting (of other kinds) and online gambling [14].

In comparison to general population adults, college-aged students may be at risk of (or at least more likely to) experiencing problem gambling disorders, especially if they are interested in sports or sports gambling [1,4,14]. Due to the long-lasting negative consequences of the problem or pathological gambling, it is important to understand what influences college students’ sports gambling intention and behavior. Accordingly, the current study used the theory of planned behavior as a theoretical framework [10].

The theory of planned behavior (TPB) has been applied to understand behavioral intention and behavior, including gambling behavior. While there are a sizable number of studies on college students’ gambling using TPB [16], not many researchers have investigated the issue of problem gambling in the context of sport-related betting and gambling [17]. Thus, the current study evaluated what predict college students’ sports gambling behavior, using TPB determinants, i.e., subjective norms (SN), attitudes towards sports gambling (ATSB), and perceived behavioral control (PBC). The following section discussed how TPB had been applied to (sport) gambling behavior [10,18].

### 1.2. Theory of Planned Behavior

TPB posits that individuals’ behavior is predicted by their behavioral intentions and perceived behavioral control, while their behavioral intentions are driven by their attitudes toward the behavior, subjective norms, and perceived control over the behavior [10]. According to the theory, gamblers’ behavioral intention is shaped by their general feelings of (un)favorableness about participating in (sport) gambling, their perceptions of whether people important to them think they should play gambling activities, and their perceived ease of playing gambling activities. In turn, their behavioral intention of gambling, which is a function of three TPB determinants, would lead to gambling activities’ actual participation.

In general population contexts, using TPB as a theoretical framework, Flack and Morris found that gambling frequency is positively associated with normative beliefs and PBC (or gamblers’ beliefs), but not with outcome expectancies [19]. In a study with Spanish adolescents (aged 12–20 years), León-Jariego et al. found that gamblers’ intention to gamble was moderately and almost equally associated with gambling attitude, the subjective norms of gambling, and gambling self-efficacy [20]. On the other hand, non-gamblers’ intention to gamble was associated with subjective norms, followed by gambling self-efficacy and gambling attitudes. In comparison to non-gamblers’ intention to gamble, adolescent gamblers’ intention was better predicted by their TPB determinant variables (32.4% of the variances in problem gamblers’ intention was explained, in comparison to 16.2% of non-gamblers’ intention). Overall, León-Jariego et al. concluded that TPB is a useful framework in understanding gambling intention and frequency in adolescent gamblers [20].

With a sample of general adults (aged 18–85 years) in Australia, Hing et al. found that subjective norms about sports betting predicted sports betting intentions, but not by their attitudes towards and behavioral control about sports betting [17]. Their study also asserted that the sports betting intention was positively related to individual Problem Gambling Severity Index (PGSI) score, previous (sports) gambling experience, exposure to gambling promotions during televised sport, and attitude toward sport-embedded gambling promotion. Given that young men are more likely to be exposed to sports-embedded gambling promotion and feel pressured to gamble to avoid social isolation from peers, a precautionary approach is suggested to develop better public health policy and interventions to minimize the negative consequences of sports gambling and sports-embedded gambling promotion [17,21].

In college-aged population contexts, Martin et al. investigated college students’ gambling behavior in the past year using the TPB and found that the TPB explained 25–30% of the variability in past-year gambling and gambling frequency [18]. Past-year gambling was predicted by friend/family norms (not peer norms) and attitudes toward gambling. In contrast, gambling frequency was predicted by friend and family subjective norms, attitudes toward gambling, and PBC, in that order. Regarding the role of social norms on college student gambling, Larimer and Neighbors found that college students’ social norms are significantly related to self-reported gambling frequency, gambling expenditure, and negative consequences of gambling problems, suggesting the critical role of social influence in developing prevention and treatment programs and norm-based social marketing campaigns [4]. Moore and Ohtsuka attempted to understand college students’ intention to gamble and problem gambling using two of the three TPB variables, namely subjective norms and attitudes [22]. Their study found that attitudes predicted intention to gamble, but not the subjective norms, while problem gambling was largely predicted by intentions. More recently, Dahl et al. found that attitudes and subjective norms best predicted intentions to gamble, i.e., operationalized by perceived normative pressure [23].

Overall, the literature suggests that the determinants of college students’ sports gambling behavior have been much less studied, despite more significant negative consequences of sport gambling addictions. The literature also suggests that TPB is an appropriate theoretical framework that can predict behaviors in both general and sport gambling domains. However, TPB has been relatively less explored in the sport gambling contexts [24]. More importantly, there has been a lack of scholarly attention on the moderating role of the problem and/or at-risk gambling severity on the relationships between TPB determinants and sport gambling intentions. For example, Hing et al. investigated sport betting intention among adults using the theory of reasoned action (TRA) and problem gambling status [17]. However, their study did not include one of the critical variables, namely perceived behavioral control (PBC), and used the problem gambling status as a grouping variable to compare the differences among groups in descriptive statistics.

On the other hand, the current study aimed to investigate the differences in the parameters (i.e., regression coefficients) across groups while including an additional variable, namely PBC. Consequently, we may find what influences college students’ sports gambling intention across recreational and problem gamblers. Therefore, the current study investigated college students’ sports betting intention and behavior using TPB. In particular, the study investigated the moderation of problem gambling severity on the relationships between study variables.

### 1.3. Research Model and Hypotheses

**Figure 1:**
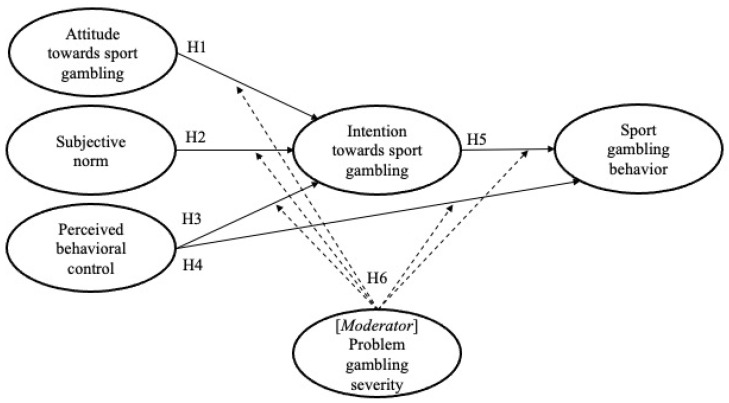
Research model with hypotheses.

As the TPB postulates, it was hypothesized that sports gambling intention and gambling behavior would be significantly associated with college students’ attitudes toward sports gambling, perceptions of family, friends, peers’ attitudes towards gambling, and perceived behavioral control of sports gambling [5,10,18,23]. It was also hypothesized that college students’ sports gambling intention would mediate the relationship between TPB determinants and sports gambling behavior [5,23]. In addition, the relative influence of TPB determinants on sports gambling intention would be moderated by the severity of problem gambling. For example, a college student with a higher level of problem gambling severity might be surrounded by family or friends who also frequently play gambling activities and, consequently, their intention to gamble is likely to be more strongly influenced by social norms in comparison to college students with a lower level of problem gambling [4]. The following hypotheses were tested in this study (also see Figure 1).

**Hypothesis** **1 (H1).** *Attitude toward sport gambling will be positively associated with a college student’s behavioral intention towards sport gambling.*

**Hypothesis** **2 (H2).** *Subjective norms toward sport gambling will be positively associated with a college student’s behavioral intention towards sport gambling*.

**Hypothesis** **3 (H3).** *Perceived behavioral control will be positively associated with a college student’s behavioral intention towards sport gambling*.

**Hypothesis** **4 (H4).** *Perceived behavioral control will be positively associated with a college student’s sport gambling behavior.*

**Hypothesis** **5 (H5).** *Behavioral intention towards sport gambling will be positively related to the intended behavior, in this case, sport gambling*.

**Hypothesis** **6 (H6).** *College students’ individual levels of problem gambling will moderate the influences of TPB determinants on behavioral intention and intended behavior towards sport gambling.*

## 2. Materials and Methods

### 2.1. Participants and Procedures

Data were collected from 334 college students from four mid-sized or large universities in the U.S., using a survey questionnaire. Of the 334 respondents, the majority were males (*n* = 226, 67.7%) and Caucasian Americans (*n* = 275, 82.3%). The average age of the participants was about 21 years old (*SD* = 2.37). The vast majority of the participants experienced some forms of gambling activities (*n* = 271, 81.1%) or sports gambling (*n* = 243, 72.8%). An ad-hoc analysis found no significant differences based on the respondents’ university affiliations, and, thus, all data were pooled for analysis.

### 2.2. Measures

The survey examined six constructs, i.e., attitude toward sports gambling (ATSG), subjective norms (SN), perceived behavioral control (PBC), sports gambling intention (SGI), sports gambling behavior (SGB), and problem gambling (PG). A 7-point Likert-type scale, ranging from strongly disagree (1) to strongly agree (7), was used. The items were taken from the previous TPB studies, and they were slightly modified to fit the context of sports gambling [10,18,20]; ATSG (five items), SN (three items); PBC (three items), SGI (two items), SGB (two items), and PG (four items). Examples of TPB items include: “I found sport gambling interesting and enjoyable” (ATSG), “people important to me think that it is okay for me to gamble on sports” (SN), “I feel that I have complete control over gambling on sports” (PBC), “I intend to continue betting on sports in the future” (SGI), “I plan to frequently participate in sport gambling activities” (SGB), and “on occasions, I have borrowed money to gamble secretly from family or friends” (PG).

### 2.3. Statistical Analysis

Initially, a preliminary test was performed to test the data’s normality using skewness and kurtosis statistics. The results revealed no major issue in terms of both skewness statistics (within the ±1.00 cut-off value) and kurtosis values of all items (smaller than 3) [24,25]. Therefore, the results supported the normality assumption required for confirmatory factor analysis (CFA) and structural equation modeling (SEM) [26]. Data were primarily analyzed using a two-step structural equation modeling approach using partial least squares structural equation modeling, aka., PLS-SEM [27]. The measurement model was assessed to estimate the reliability and validity of the scales used in this study. Subsequently, the structural model was tested to the hypotheses and examine the relationships between study variables. Lastly, a multi-group analysis was conducted to explore the differences between lower-risk (recreational/social) and higher-risk (problem) gamblers.

## 3. Results

### 3.1. Measurement Model Assessment

The initial measurement model yielded an acceptable fit for the data [28]. However, two items were removed due to the low factor loadings. The revised measurement model fitted the data well, *x*^2^ = 316.06, *df* = 120, *x*^2^/*df* = 2.63, CFI = 0.955, *p* < 0.001, CFI = 0.94, TLI = 0.92, RMSEA = 0.070.

Table 1 reports the correlations between study variables as well as the reliability and validity results. The reliability of the measures was calculated using Cronbach’s alpha and composite reliability (CR). In contrast, the measures’ validity was examined using the average variance extracted (AVE) and factor loadings. All values exceeded the recommended threshold of Cronbach’s *α* (0.70), AVE (0.50), CR (0.70), and factor loading (0.50), except a Cronbach’s α value of PBC [29,30,31]. However, many researchers, such as DeVellis and Vaske, also suggested that a Cronbach’s α above 0.65 is adequate as an internal consistency indicator [32,33]. It should also be pointed out that the PBC scale used in this study has been empirically established and tested for reliability and validity in previous studies. Therefore, the measurement model and measures were used without further modification.

In terms of the correlations between the three TPB predictors, the highest correlation was found between attitude and subjective norms (*r* = 0.55, *p* < 0.001), followed by attitude and PBC (*r* = 0.27, *p* < 0.001) and subjective norms and PBC (*r* = 0.25, *p* < 0.001).

### 3.2. Structural Model PLS-SEM Analysis

The structural model yielded an adequate level of fit, chi-square = 207.53, *df* = 69, CMIN = 3.01, CFI = 0.95, TLI = 0.93, RMSEA = 0.078 (see Table 2). The model explained substantial amounts of variances in sport gambling intention (46.1%) and sport betting behavior (51.2%). Of the three TPB predictor variables, the intention to bet on sports was most highly predicted by attitude (*β* = 0.49, *p* < 0.001), followed by subjective norms (*β* = 0.29, *p* < 0.001), but not by PBC, accepting H1 and H2, but rejecting H3. Sport betting behavior was predicted by the intention (*β* = 0.70, *p* < 0.001) and PBC (*p* = 0.09, *p* = 0.02), accepting H4 and H5. Overall, the results supported the basic tenets of TPB in the context of sport betting, except the relationship between TPB and betting behavior.

As reported in Table 2, we also conducted mediation analyses to investigate the significance of indirect effects. There were significant effects of attitude and subjective norms via intention on sports betting behavior (*β* = 0.34, *p* < 0.001 and *β* = 0.20, *p* < 0.001, respectively), meaning that both attitudes and subjective norms, both directly and indirectly, influence college students’ sports betting behavior. However, we did perceive behavioral control via intention on sports betting behavior was not statistically significant.

### 3.3. Multi-Group Analysis

Furthermore, multi-group PLS-SEM was conducted to investigate the moderating role of gambling addiction. Based on the level of problem gambling severity, the sample was split into two groups: lower-risk (recreational) gamblers (*n* = 217) and higher-risk (problem) gamblers (*n* = 117). Therefore, cluster analysis was conducted on participants’ problem gambling severity scores. The average composite PG scores were 4.32 for the lower-risk gambler group and 12.33 for the higher-risk gambling group, suggesting a substantial difference between the two groups on problem gambling severity scores (*p* < 0.001). In the case of lower-risk gamblers, their sport gambling intention was mostly predicted by attitude (*β* = 0.47, *p* < 0.001), followed by subjective norms (*β* = 0.23, *p* < 0.001), but not by PBC. Sport betting behavior was predicted by the intention (*β* = 0.68, *p* < 0.001) and PBC (*β* = 0.11, *p* = 0.016). In the case of higher-risk gamblers, their sport betting intention was predicted by subjective norms (*β* = 0.42, *p* < 0.001) and attitudes (*β* = 0.32, *p* = 0.004), but not by PBC. Problem gamblers’ sport betting behavior was predicted by both the intention (*β* = 0.46, *p* < 0.001) and PBC (*β* = 0.17, *p* = 0.016).

In addition, we also conducted mediation analyses per group (see the bottom part of Table 3). For both groups, the indirect effects of attitude and subjective norms via intention on behavior were significant, while no indirect effect of PBC via intention on behavior was found. Regarding group differences, a statistically significant difference was found in the indirect effect of attitude via intention on sport gambling behavior (*p* = 0.041) between the two groups, indicating that the role of ATSG is more influential in predicting lower-risk gamblers’ behavior. Overall, the results partially supported H6.

## 4. Discussion

Extant literature has paid relatively little attention to college students’ sports gambling behavior and the role of problem gambling severity in their decision-making process in the context of sports gambling. Accordingly, the current study investigated what determines college students’ sports gambling behavior using the TPB and investigated the moderating role of problem gambling severity on TPB variables’ relationship. Overall, the results found that sports gambling intention was better predicted by attitude, followed by subjective norms. In contrast, sports gambling behavior was best predicted by intention, along with perceived behavioral control. The study also found some interesting differences between lower-risk (recreational) and higher-risk (problem) gamblers. Lower-risk gamblers’ sports gambling intention was better predicted by their attitude towards sports gambling, followed by their subjective norms, while higher-risk gamblers’ intention to gamble on sport was best predicted by their subjective norms, followed by their attitude. Also, the relationship between intention and behavior was more robust among recreational sports gamblers. Implications of these findings are discussed below.

### 4.1. Attitude Formation or Change

Of the three TPB determinants, attitude towards sports gambling was the most significant antecedent of behavioral intention, and it also indirectly predicted college students’ sports gambling behavior, highlighting the importance of attitude formation and change concerning sports gambling among the college-aged populations [17,22]. As noted earlier, sports gambling is not necessarily an undesirable activity, and thus, college students, especially social and recreational gamblers, do not need to have a negative attitude towards sports gambling. However, college students should not have an overly positive attitude towards sports gambling and should know the potential negative consequences of excessive sport gambling activities. Problem gamblers have a strong desire to win money and consider gambling an income-generating activity [34,35]. Thus, education programs might be more effective if the programs can effectively educate college students on the primary purpose of sports gambling (i.e., an entertainment activity, not an income-generation activity) and the accurate estimate of the likelihood of winning [35,36].

According to social learning and exposure effects [37,38], the media influences our attitude and behavior, and thus, continuous exposures to gambling-related media messages can cultivate unhealthy behavior [17,39]. The proliferation of gambling promotions during televised sports events (i.e., sports-embedded gambling promotions), especially sports gambling promotions during sports events, can be seen as an agent of attitude formation, especially for young men as adolescents and college-aged students [39]. Many gambling and sport betting companies try to put their names to the mass and social media by becoming corporate sponsors for sports entities, such as professional leagues and sports tournaments [39]. While each country and/or sports entity has different policies and regulations concerning sport-embedded gambling promotions and corporate sponsorships in sports, it is clear that the decision-makers should think about the possible negative influence of such practices on young adults [39], given the media’s role in influencing college students’ attitude towards sports gambling.

### 4.2. Subjective Norms

The results suggest that people important to (or around) college students, such as parents, relatives, friends, and peers, significantly influence their intention towards sports gambling. Parental and peer influences can prevent them from engaging in excessive sports gambling behavior by influencing their intention [4,18]. From a different angle, college students are likely to have a higher behavioral intention towards sports gambling and participate in sport gambling activities if their parents or peers play or endorse sports gambling. Thus, significant others’ approval or disapproval of sports gambling is a critical facilitator or regulator of college students’ sports gambling intention.

Regarding parental influences, health preventions and education programs should encourage parents to engage in their college-bound children’s responsible gambling education by demonstrating clear parental norms and being a role model. College students are still significantly influenced by their friends and peers, and thus, having friends who are into sports gambling has a strong influence on their sports gambling behavior. Some of the main reasons college students’ involvement in sports gambling include peer competitiveness, social pressure, and proving manhood/sisterhood through gambling [40]. Consequently, the primary target of gambling prevention and treatment should be college students who are surrounded by frequent sports gamblers or who have overestimated perceptions of peer gambling and peer approval of sports gambling. Prevention and treatment programs should focus on changing such misperceptions (i.e., subjective norms) and handling peer pressure for sports gambling [4].

### 4.3. Problem Gambling Severity

In the case of recreational (non-problem) sport gamblers, among the three TPB determinants, college students’ attitude was the most significant predictor of their sports gambling intention, followed by the subjective norms and perceived behavioral control. Consequently, attitude indirectly predicted their gambling behavior. On the other hand, college students’ sport gambling intention with a higher level of problem gambling severity was greatly influenced by subjective norms compared to their lower-risk, recreational gamblers.

According to Nower and Blaszczynski [34], when playing electronic gaming machines, problem gamblers are motivated to get away from daily hassles and issues, to earn additional income, and to consume the exciting and entertaining aspects of gambling. In contrast, non-problem gamblers play gambling for fun and enjoyment [34]. Thus, prevention and treatment programs should consider individual differences in problem gambling severity and such differing gambling motivations when developing responsible gambling strategies. Especially for students with a higher severity level of problem gambling, subjective norms toward sports gambling were more influential in predicting their intention and behavior. Consequently, problem-gambling prevention initiatives should incorporate ways to build college students’ developmental competencies, such as cognitive competence and self-efficacy, concerning sports gambling and other gambling activities. These will help them understand that they can have self-perceptions of invulnerability and control their behavior towards sports gambling [24].

Furthermore, the relationship between intention and behavior towards sports gambling was more robust with the lower-risk, recreational gambling group than their higher-risk, problem-gambling counterpart. Given the nature of problem gambling, it is expected that the behavior of college students with severe problem gambling issues would be more driven by addiction and impulsivity. Unlike the TPB’s proposition, the current study failed to find a strong relationship between perceived behavioral control and intention, probably given the nature of the study context and recent development in sport gambling technologies. In other words, prevention and treatment programs on problem gambling should focus on changing attitude formation and providing social support to those suffering from gambling addiction [24].

### 4.4. Limitations

While the current study provides some valuable information on college students’ sports gambling behavior concerning problem gambling, there are some noteworthy limitations. As noted, the current study used the TPB as a theoretical framework and thus did not include extended variables, such as materialism and impulsivity [14]. In addition, the current study used a cross-sectional design and may not provide an actual cause-effect relationship between study variables. Thus, further studies may consider plausible extended variables and different research designs to investigate college students’ sports gambling behavior.

## 5. Conclusions

Due to the increased legalization of sports gambling in the U.S., there is a growing need to understand what influences college-aged students’ sports gambling behavior. The current study results suggest that forming a responsible attitude and subjective norms towards sports gambling and having greater perceived behavioral control are important antecedents of sports gambling intention and behavior. Health prevention and education programs should first direct their efforts to form a responsible attitude towards sports gambling, given the relative influence of attitude on intention and behavior. To avoid the negative consequences of problem gambling among college students, public awareness campaigns (e.g., responsible gambling messages) should be designed with this specific audience in mind. College students with severe problem gambling activities are likely to be more influenced by their subjective norms, while their low-risk counterparts are greatly influenced by attitude. Consequently, the health and education agencies should consider such differences when designing prevention and treatment programs for problem gambling on sports.

## Figures and Tables

**Table 1:** Correlations, reliability and validity.

Variable	1	2	3	4	5	Cronbach’s *α*	AVE	CR
1. Attitude						0.84	0.61	0.89
2. Subjective norms	0.55 ***					0.80	0.71	0.88
3. PBC	0.27 ***	0.25 ***				0.67	0.74	0.85
4. Intention	0.64 ***	0.54 ***	0.15 **			0.88	0.89	0.94
5. Behavior	0.62 ***	0.47 ***	0.19 **	0.71 ***		0.92	0.93	0.96
6. Problem gambling	0.42 ***	0.31 ***	−0.01	0.60 ***	0.49 ***	0.82	0.53	0.81

Note: PBC = Perceived behavioral control; AVE = average variance extracted; CR = composite reliability; ** *p* < 0.01, *** *p* < 0.001.

**Table 2:** Results of the hypothesized model.

Direct/Indirect Paths	*β*	Standard Error	*t*-Value
Hypothesized paths			
H1. Attitude → Intention	0.49	0.053	9.72 ***
H2. SN → Intention	0.29	0.054	5.34 ***
H3. PBC → Intention	−0.05	0.045	1.18
H4. PBC → Behavior	0.09	0.037	2.33 *
H5. Intention → Behavior	0.70	0.032	21.93 ***
Specific indirect effects			
Attitude → Intention → Behavior	0.34	0.044	7.81 ***
SN → Intention → Behavior	0.20	0.038	5.32 ***
PBC → Intention → Behavior	−0.04	0.030	1.19

Note: SN = Subjective norms; PBC = Perceived behavioral control; * *p* < 0.05, *** *p* < 0.001.

**Table 3:** Group differences: Direct and indirect effects.

Direct/Indirect Paths	Low PGS (*n* = 217)	High PGS (*n* = 117)	Significant Difference
	*β*	*β*	*p*-Value
Direct paths			
Attitude → Intention	0.47 ***	0.32 ***	0.243
SN → Intention	0.23 ***	0.42 ***	0.108
PBC → Intention	−0.03	−0.01	0.842
PBC → Behavior	0.11 *	0.15	0.569
Intention → Behavior	0.68 ***	0.46 ***	0.003 **
Indirect paths			
Attitude → Intention → Behavior	0.32 ***	0.15 *	0.041 *
SN → Intention → Behavior	0.16 ***	0.19 ***	0.625
PBC → Intention → Behavior	−0.02	0.00	0.800

Note: PGS = Problem gambling severity; SN = Subjective norms; PBC = Perceived behavioral control; * *p* < 0.05, ** *p* < 0.01, *** *p* < 0.001.

## Data Availability

The data presented in this study are available on request from the corresponding author. The data are not publicly available due to privacy issues.
